# Metabolic and Inflammatory Profiles in Children and Adolescents with Autism Spectrum Disorder: A Cross-Sectional Study

**DOI:** 10.3390/brainsci14111052

**Published:** 2024-10-24

**Authors:** Joana M. Gaspar, José Pedro Ferreira, Humberto M. Carvalho, Chrystiane V. A. Toscano

**Affiliations:** 1 Laboratory of Neuroimmune-Metabolism, Federal University of Santa Catarina, Florianópolis 88037-000, SC, Brazil; 2 Graduate Program in Biochemistry, Federal University of Santa Catarina, Florianópolis 88037-000, SC, Brazil; 3 Faculty of Sport Sciences and Physical Education, University of Coimbra, 3004-531 Coimbra, Portugal; jpferreira@fcdef.uc.pt; 4 Department of Physical Education, School of Sports, Federal University of Santa Catarina, Florianópolis 88040-900, SC, Brazil; hmoreiracarvalho@gmail.com; 5 Institute of Physical Education and Sport, Federal University of Alagoas, Maceió 57072-970, AL, Brazil

**Keywords:** autism, obesity, insulin resistance, dyslipidemia, immune dysfunction

## Abstract

Background/Objectives: Autism spectrum disorder (ASD) is associated with several coexisting diseases or comorbidities, including inflammatory and metabolic disorders. In fact, ASD symptoms may be associated with immune system dysfunction. However, studies investigating the peripheral blood levels of immune cells are lacking and have provided mixed findings. In this study, we evaluated the relationship between the intensity level of ASD symptoms and the inflammatory and metabolic profiles in 154 children and adolescents (2–17 years). Methods: Bayesian multilevel models were used to examine the relationship between their symptom intensities and inflammatory/metabolic profiles. Results: Heavier children had higher values for triglyceride and insulin levels. Children with a level 3 of ASD intensity had higher free fatty acids levels. However, when adjusting for ASD intensity, gender, medication use, or weight status, older children appeared to have higher values of triglycerides, insulin levels, and free fatty acids. Conclusions: We concluded that as Brazilian children with ASD became older, they had a higher risk for insulin resistance.

## 1. Introduction

Autism spectrum disorder is a complex neurodevelopmental disorder characterized by the following primary symptoms: (1) deficits in social communication and interaction and (2) repetitive patterns of behavior and restricted interests in activities [1]. The etiology of the disorder is not clear yet, although there is consensus about the contribution of genetic and environmental factors to ASD development [2,3,4,5]. ASD affects 1 in 36 children in the United States of America, occurring in all racial, ethnic, and socioeconomic groups [6]. Globally, autism has a median prevalence of 100/10,000 [7]. In Brazil, in 2010, it was estimated that approximately 500,000 individuals had autism, which represents around 0.3% of the population [8].

ASD is associated with several coexisting diseases or comorbidities. Metabolic disorders, inflammatory diseases, gastrointestinal disorders, obesity, and limitations in motor activity, are some of the most common comorbidities in populations with ASD [2,3,9,10]. In addition, the cumulative effect of primary symptoms, diseases, or comorbidities associated with ASD, as well as continuous drug use, seem to make populations with ASD more vulnerable to weight gain and metabolic disorders [11,12,13]. Also, evidence suggests that ASD symptoms may be associated with immune system dysfunction [14,15,16,17]. However, studies investigating the peripheral blood levels of immune cells are lacking and have provided mixed findings. In a meta-analysis study, it was observed that individuals with ASD showed significantly higher levels of white blood cells, neutrophils, and monocytes compared to typically developing controls. However, no differences were found with respect to the levels of lymphocytes and B cells [18], suggesting that ASD individuals might have abnormal levels of immune cells. Nevertheless, larger longitudinal studies including potential confounding factors such as body mass index and metabolic markers are needed.

Children with ASD have an increased risk of obesity and obesity-related metabolic disorders compared with healthy children [19,20,21,22,23]. For example, it was reported that in children with ASD, the prevalence of overweight was 42.4% and 21.4% for obesity, compared to 26.1% for overweight and 12.0% for obesity among controls [24]. Associated with the increase in obesity prevalence, this population also had an increased prevalence of developing metabolic syndrome, with 31.5% for dyslipidemia and 19.4% for hypertension and type 2 diabetes [19].

Children with ASD may also have gastrointestinal disturbances, feeding problems [25,26] associated with disruptive behaviors, and selective eating behaviors that can lead to obesity. The increased prevalence of obesity among children with ASD can occur due to their unusual dietary patterns, decreased access to opportunities for physical activity (motor and social skill deficits), and also because of prescribed psychotropic medications [9,11,20,21,27].

Between 46% and 89% of children with ASD exhibit nutritional challenges and frequently have selective eating behaviors manifested by choosing more energy-dense foods. For example, children with ASD reportedly ate fewer vegetables, salads, and fresh fruit and ate more energy-dense foods [28,29,30].

The present study examined the relationship between the levels of symptom intensity of autism spectrum disorder and the inflammatory and metabolic profiles of Brazilian children and adolescents aged from 2 to 17 years. In addition, we adjusted for age, weight, and pharmacological medication.

## 2. Materials and Methods

### 2.1. Design and Participants

This study used a cross-sectional design. Children and adolescents were recruited from a specialized pediatric center (CUIDA) for populations with ASD located in Maceió, Alagoas (Brazil), and were aged 2–17 years (n = 154). The chronological age was calculated to the nearest 0.1 years as birth date minus assessment date and later categorized into age groups (2–5, 6–9, 10–13, and 14–17 years). Research recipients using or not using drugs were identified from the clinical records stored in the specialized pediatric center.

The inclusion and recruitment criteria were as follows: to have a certified medical diagnosis of ASD following the standards established in the DSM-5 [1]; to present a recent history of non-participation in physical activity programs or similar physical-motor activities; and an absence of other syndromes associated with ASD. In addition, the study was carried out following the ethical standards of the Declaration of Helsinki and approved by the Human Ethics Committee of the Federal University of Alagoas (Protocol number 1.091.864 and CAEE number: 41286815.0.0000.5013). In all cases, their families received information about the study and signed an informed consent form.

### 2.2. Intensity of the ASD

The intensity of symptoms was identified from clinical records stored at the CUIDA for populations with ASD. The multidisciplinary team of the specialized service uses the criteria of the DSM-V for classifying the level of intensity of ASD symptoms. These are related to the volume of specialized support that the child needs: (a) Level 1 (n = 104): subjects need specialized support; this category includes individuals who without individual support demonstrate deficits in social communication that imply relevant damage, demonstrate restricted and repetitive behavior, and have inflexible behaviors that affect their functioning in the most diverse contexts. (b) Level 2 (n = 36): subjects need substantial specialized support; this category includes individuals who without support have severe deficits in verbal and non-verbal social communication, restricted and repetitive behavior and behavioral inflexibility, difficulty in dealing with changes, and interference in the functioning of a variety of contexts. (c) Level 3 (n = 14): subjects need highly specialized support; they have severe deficits in verbal and non-verbal social communication skills that significantly impair their functioning, causing significant limitations in starting social interactions due to their minimal response to the social openings of others [1].

### 2.3. Anthropometry

Stature was measured with a portable stadiometer (Seca model 206, Hanover, MD, USA) to the nearest 0.1 cm. Body mass (BM) was measured with a calibrated portable balance (Seca model 770, Hanover, MD, USA) to the nearest 0.1 kg. The body mass index (BMI) was calculated as body mass (kg) divided by the squared stature (m). The BMI was used to classify children and adolescents into the following four categories: obese (body mass index ≥ 95th percentiles), overweight (≥85th to <95th percentiles), healthy (>5th to <85th percentiles), and underweight (≤5th percentile). Chronological age was calculated to the nearest 0.1 years as birth date minus testing date. Waist circumference was measured in centimeters using a non-extendable flexible tape with a precision of 0.1 cm. The tape was applied above the iliac crest, parallel to the ground, with the individual standing with their abdomen relaxed, arms alongside the body, and feet together. All assessments were made by a single experienced observer [31].

### 2.4. Blood Sample and Analysis

Blood collections were taken at the specialized pediatric center for populations with ASD by a health professional (nurse) experienced in collecting blood from children with ASD. Blood samples were collected after a 12 h overnight fast by venipuncture, and collected in K_3_EDTA tubes (Labor Import, São Paulo, Brazil). Blood samples were analyzed for complete blood cell counts, glucose, total cholesterol, High-Density Lipoprotein Cholesterol (HDL-C), Low-Density Lipoprotein Cholesterol (LDL-C), fatty acids, and triglycerides, using a standard protocol [32].

A white blood cell count was performed using an automated hematology instrument MINDRAY BC 6800 (Mindray, Shenzhen, China) [33]. The measurement of white blood cells was performed using the differential blood count method. Glucose, total cholesterol, HDL-C, and triglycerides were measured using the ADVIA 1800 instrument (Siemens, Berlim, Germany). LDL-C was calculated using Friedewald’s formula [34], and glucose was measured by an enzymatic method (Glucose Oxidase—Lab test). A Non-esterified Free Fatty Acids (NEFA/FFA) Colorimetric Assay Kit (Thermo Fisher Scientific, Waltham, MA, USA) was used and measured using a Microplate Reader (Infinite M200 TECAN, Life Sciences, Männedorf, Switzerland). The total cholesterol was determined using the enzymatic colorimetric method (VIDA biotecnologia, Belo Horizonte, Brazil). Insulin was measured by immunoassay using the instrument ADVIA Centaur XP (Siemens, Germany). The IRI calibrator, IRI Diluent and Insulin Master Curve used were the original matching reagents from Siemens, and the Lyphochek Immunoassay Plus Control was from Bio-Rad Laboratories (Hercules, CA, USA). The Homeostatic Model Assessment for Insulin Resistance (HOMA-IR) value was calculated according to the following formula: HOMA-IR = fasting serum glucose (mg/dL) × fasting insulin (mIU/L)/405 [35]. All the analyses were performed in a certified laboratory of clinical analysis (Clinilab, Laboratório de Análises Clínicas, Macieó, AL, Brazil).

### 2.5. Statistical Analysis

Our estimations were based on Bayesian hierarchical models [36], considering the variation in inflammatory and metabolic profiles, adjusting for cross-classified nesting by age group, gender, the intensity of the ASD, medication, and weight status among the children and adolescents with ASD. For interpretative convenience and computational efficiency, the outcomes were standardized, dividing each score by the grand mean and dividing by the sample’s standard deviation. We used varying intercept models where each participant’s outcome (intercept) was estimated as a function of their age group, gender, ASD intensity, medication, and weight status. Hence, for participant i, the indexes a, g, d, m, and w represented age group, gender, ASD intensity, medication, and weight status, respectively. The group-level effect terms (often referred to as random effects) and data-level terms (often referred to as level-1 residuals) were drawn from normal distributions with variances to be estimated from the data:

$$y_{i}=\beta^{0}+\alpha_{a\left[i\right]}^{age\;group}+\alpha_{g\left[i\right]}^{gender}+\alpha_{d\left[i\right]}^{intensity\;of\;ASD}+\alpha_{m\left[i\right]}^{medication}+\alpha_{w\left[i\right]}^{weight\;status}+\epsilon_{i}$$

$$\alpha_{a\left[i\right]}^{age\;group}\sim N\;(0,\sigma_{age\;group}^{2}),\;for\;a=1,2,3,4.$$

$$\alpha_{g\left[i\right]}^{gender}\sim N\;(0,\sigma_{gender}^{2}),\;for\;g=1,2.$$

$$\alpha_{d\left[i\right]}^{intensity\;of\;ASD}\sim N\;\left(0,\sigma_{intensity\;of\;ASD}^{2}\right),\;for\;d=1,2,3.$$

$$\alpha_{m\left[i\right]}^{medication}\sim N\;(0,\sigma_{medication}^{2}),\;for\;m=1,2.$$

$$\alpha_{w\left[i\right]}^{weight\;status}\sim N\;\left(0,\sigma_{weight\;status}^{2}\right),\;for\;w=1,2,3,4.$$

$$\epsilon_{i}\sim N\;(0,\sigma_{y_{i}}^{2})$$

We used weakly informative priors to regularize our estimates, including a normal prior (0,5) for the intercept (a population-level parameter often referred to as a fixed effect) and a half-normal prior (0,1) for the group-level parameters. We used the “brms” default prior, Student-t (3, 0, 2.5), for the data-level residuals (ϵi). Given the standardized outcomes in the models and using a normal (0,1) prior for the parameters, we state that the group-level estimates are unlikely to be greater than one standard deviation. We ran four chains for 500 iterations with a warm-up length of 250 iterations in each model. The convergence of Markov chains was inspected with trace plots. We used posterior predictive checks to be confident in our models and estimations [36]. The models were fitted in R [37] using the “brms” package [38], which calls Stan [39].

## 3. Results

The characterization of the sample of the study is represented in Table 1. The distribution of children and adolescents with ASD was as follows: underweight, n = 2; normal weight, n = 64, overweight, n = 24; and obese, n = 64. The number of children and adolescents with an ASD at level 1 are 104, level 2 are 36 and level 3 are 14. The descriptive statistics of inflammatory indicators for children with ASD for the total sample are summarized in Table 2. No relevant variation was detected using Bayesian hierarchical modeling for lymphocytes, leukocytes, and monocytes when adjusted for age, ASD intensity, gender, medication use, or weight status. Also, the pediatric populations’ lymphocyte, leukocyte, and monocyte values were within the normal range (according to the CDC).

**Figure 1:**
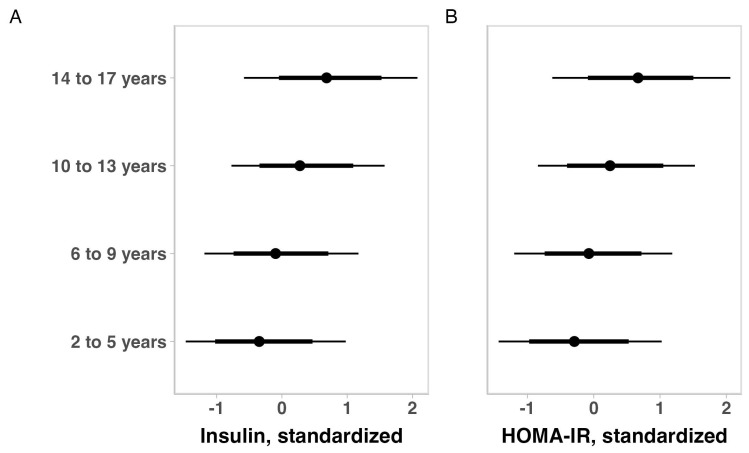
Posterior estimations and uncertainty (bold lines and thick lines represent 67% and 90% intervals, respectively) for insulin (**A**) and HOMA-IR (**B**) values (standardized) by age group.

**Figure 2:**
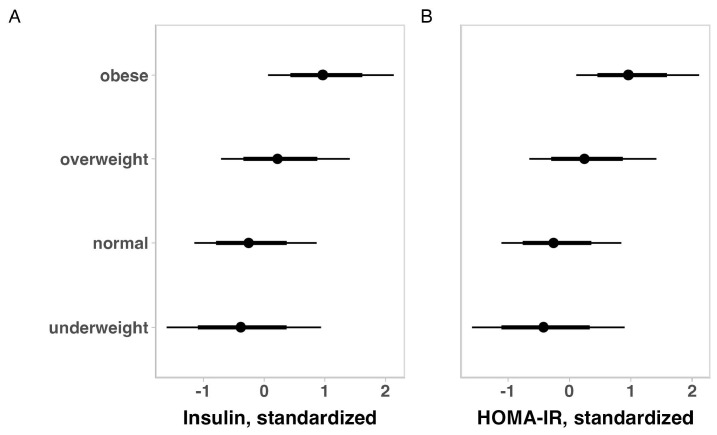
Posterior estimations and uncertainty (bold lines and thick lines represent 67% and 90% intervals, respectively) for insulin (**A**) and HOMA-IR (**B**) values (standardized) by weight status.

The metabolic indicators of children with ASD for the total sample are summarized in Table 3. No substantial variation was observed for glycemia, insulin, and HOMA-IR when the outcomes were adjusted for ASD intensity, gender, and medication use. When adjusting for ASD intensity, gender, medication use, or weight status, older children appeared to have higher insulin and HOMA-IR values (Figure 1). Also, adjusting for age, ASD severity, gender, and medication use, revealed that heavier children seem to have higher insulin and HOMA-IR levels (Figure 2).

**Figure 3:**
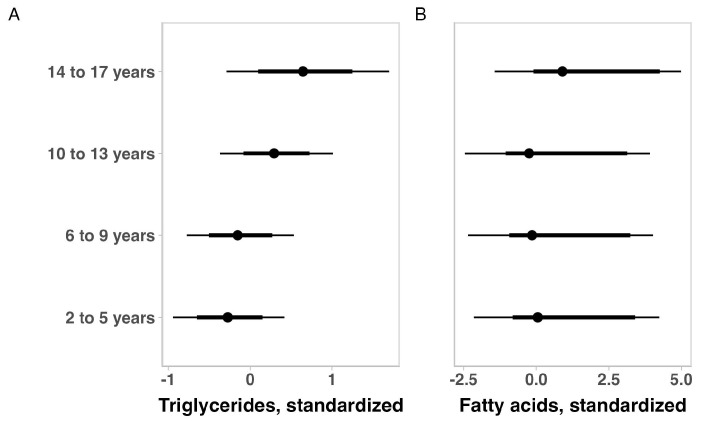
Posterior estimations and uncertainty (bold lines and thick lines represent 67% and 90% intervals, respectively) for triglyceride (**A**) and fatty acid (**B**) values (standardized) by age group.

**Figure 4:**
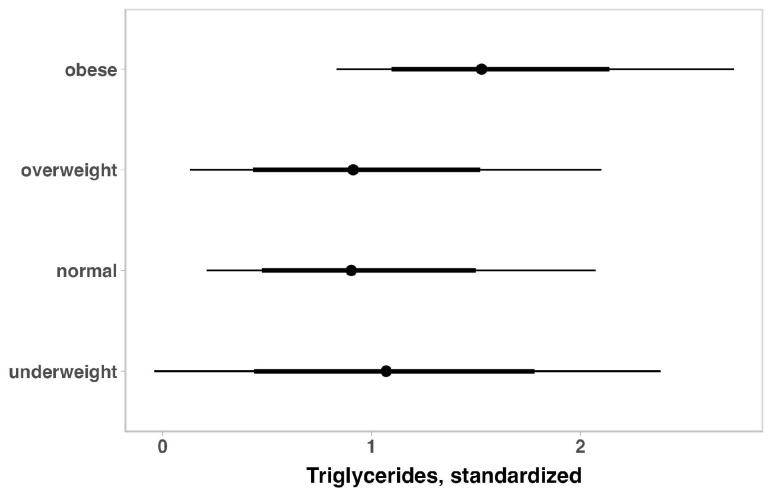
Posterior estimations and uncertainty (bold lines and thick lines represent 67% and 90% intervals, respectively) for triglyceride values (standardized) by weight status.

**Figure 5:**
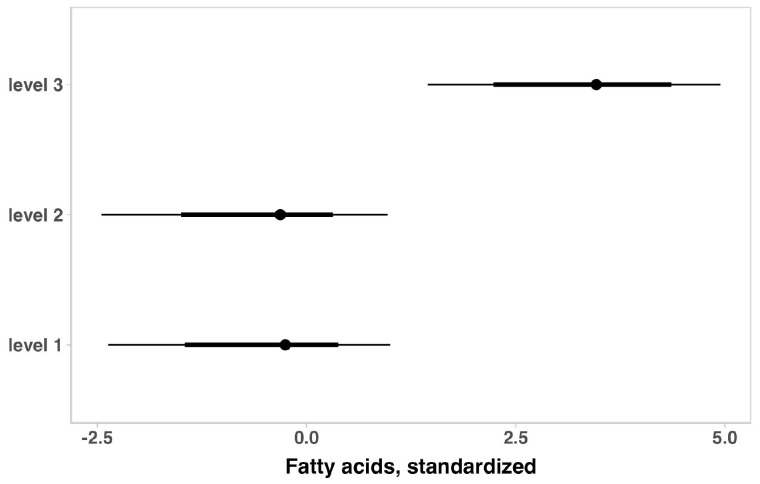
Posterior estimations and uncertainty (bold lines and thick lines represent 67% and 90% intervals, respectively) for fatty acid values (standardized) by ASD intensity.

The lipid profile indicators in children with ASD for the total sample are summarized in Table 4. No substantial variation was observed for total cholesterol, HDL, and LDL when the outcomes were adjusted for age, ASD intensity, gender, medication use, or weight status. When adjusting for ASD intensity, gender, medication use, or weight status, older children appeared to have higher values of triglycerides and free fatty acids (Figure 3). On the other hand, adjusting for age, ASD severity, gender, and medication use, showed that heavier children seemed to have higher triglyceride levels (Figure 4). Considering the ASD intensity, children with level 3 ASD have substantially higher free fatty acid levels (Figure 5).

## 4. Discussion

The main conclusion of this study was that older Brazilian children with ASD have higher values for triglycerides, insulin levels, free fatty acids, and the HOMA index, indicating a higher risk for insulin resistance. However, no changes were observed for inflammatory cells. Also, the inflammatory cells and metabolic markers (triglycerides, HDL-C, LDL-C, cholesterol, and glucose) were within the normal range for the pediatric population (according to the CDC). These markers can potentially be used as good tools to follow up the management of patients with ASD from their earliest childhood.

Several studies conducted in different countries worldwide have examined the prevalence and risk of overweight and obesity development in children with ASD [11,24,27]. A recent meta-analysis [40] suggests that children with ASD seem to have a greater risk of developing overweight or obesity, particularly when living in the United States [40]. In addition, many psychiatric medications used to manage ASD symptoms may influence intermediate metabolism leading to high triglycerides, weight gain, and other dysmetabolic features that, in turn, are associated with insulin resistance. However, cultural differences have been postulated to moderate the association between ASD and excessive weight gain.

The higher prevalence of obesity in the ASD population can be explained by several factors, including the lack of engagement in physical activities [41], the dysregulated diet composition with atypical selectivity (e.g., preference for more calorically dense food) [29], and sleep disturbances [42]. Children with ASD have been shown to have deficits in motor development, motor skills, a lack of engagement in daily activities, and a decreased motivation to engage in beneficial physical activity [43]. Children and adolescents with ASD are less motivated to participate in physical activity behaviors than their typically developing peers [44]. Adolescents with ASD were more likely to be overweight or obese and less likely to engage in physical activity behaviors [41].

Our study demonstrated that free fatty acids were higher in obese individuals, primarily due to their release from the increased fat mass due to a resistance (HOMA-index) to the antilipolytic effect of insulin in obese adipocytes [45]. Increased levels of free fatty acids have been associated with endothelial dysfunction, blood pressure, and pancreatic β-cell dysfunction [46]. The data regarding cholesterol levels in the ASD population are controversial. To date, only a few studies have reported alterations in cholesterol levels associated with ASD conditions. In our study, cholesterol values were within the range for the normal population, and we did not find differences in total cholesterol, HDL-C, and LDL-C when adjusted for age, ASD intensity, gender, medication use, or weight status. Also, the HDL-C, LDL-C, and total cholesterol values were within the normal range for the normal population. However, a cross-sectional study with ASD individuals showed a high prevalence of hypocholesterolemia, with 16% showing low-density lipoprotein (LDL)-cholesterol levels below the fifth centile of the normal population [47]. Another study also showed lower HDL-C levels in individuals with ASD compared to controls [48]. Hypocholesterolemia prevalence is threefold higher in ASD individuals than in the general population [49]. Surprisingly, TG and HDL-C did not correlate with BMI in ASD-affected individuals, unlike in the controls [49,50,51].

Some evidence has suggested that ASD symptoms may be associated with an altered immune system [14,15,17,52,53]. While it would indeed be interesting to compare children with ASD to neurotypical children and adolescents, we currently do not have access to such a sample. It is also worth noting that all metabolic and inflammatory parameters in our study were within the reference range, despite several studies indicating that children with ASD often have elevated levels of inflammatory and metabolic markers. Several post-natal findings in the peripheral immune system of children with ASD were documented (for review [54]). For instance, ASD children have an altered immune cell ratio, a decreased number of T lymphocytes, and dysfunctional responses and gene expressions in peripheral blood leukocytes [55,56,57]. Nevertheless, these studies did not detect relevant variation for lymphocytes, leukocytes, and monocytes when adjusted for age, ASD intensity, gender, medication use, or weight status. We also observed that in our ASD population, the values for the immune cells analyzed were within the normal range for the pediatric population. Some studies showed an increase in monocytes in children with ASD [58,59,60], while others showed no changes. Recently, it was demonstrated that monocytes in ASD children were significantly higher than in the control population [59]. However, the reported monocyte count for children with ASD was in the normal range for pediatric populations [59]. Although our observations also showed an increased number of monocytes within the normal range for pediatric populations, we could not exclude the possibility of macrophage overactivation with the associated release of cytokines and neopterin, as previously observed in ASD children [14,60].

A negative correlation between age and the lymphocyte count and a negative relationship between ASD intensity and the number of lymphocytes has been noted [59]. Nonetheless, our study did not detect changes in the whole population of lymphocytes, and we did not differentiate the number of lymphocyte populations. Changes have been observed in the peripheral blood lymphocyte subsets, such as a reduced total number of lymphocytes, impairment of the CD4/CD8 T cell ratio, a defective activation of T cells, an imbalance of the Th1/Th2 cytokines, and the presence of autoantibodies directed toward the central nervous system (CNS) proteins, suggesting a deregulation of B lymphocytes in ASD populations [61,62]. Recently, a meta-analysis showed abnormalities in the number of peripheral CD4+ lymphocytes, especially Th17 and Treg cells, in patients with ASD [63].

### 4.1. Study Implications

ASD children are at particularly high risk for overweight, obesity, and metabolic and inflammatory diseases. Evidence suggests that autistic children and adolescents cannot meet the minimum dietary, exercise, and sleep recommendations that promote good health and prevent chronic diseases. Tracking the metabolic and immunological profile of children with ASD is beneficial for informing families and physicians in order to plan personalized therapies which aim to prevent and minimize the risk for chronic disorders. Also, to inform public health services to allocate limited resources and determine subgroups at risk for preventable chronic disorders.

### 4.2. Study Limitations

This work had some limitations. The most important was the small sample size and the lack of an age-matched control population. Therefore, this study should be reproduced using a larger sample size. In addition, this study may also have been subject to sampling bias, as we recruited participants from the same institution, which provided the same care and therapeutics to ASD children. While it would indeed be interesting to compare children with ASD to neurotypical children and adolescents, we currently do not have access to such a sample. It is also worth noting that all metabolic and inflammatory parameters in our study were within the reference range, despite several studies indicating that children with ASD often have elevated levels of inflammatory and metabolic markers [16,64]. Future studies should address the comparison with neurotypical children. In a previous study, we demonstrated that children with ASD, aged from 4 to 15 years, exhibited a significant prevalence of overweight and obesity during early childhood [13], compared to the Centers for Disease Control and Prevention data. Our sample may not have represented all autistic children, as it primarily comprised individuals who resided in Maceio/Brazil. Therefore, it is important to note that the results of this study may not be generalizable to the entire autistic population. Nevertheless, our sample represented significant group differences in ethnicity, education, and intellectual disability status distributions. Another limitation of our sample was that the majority of the participants were on medication (antidepressants, antipsychotics, antiepileptics, and stimulants), with only 34 children not using any medication. Additionally, most participants were on a combination of more than one medication, and all had been taking them since their diagnosis.

Another important limitation was the lack of information about ASD biogenesis (genetic vs. behavioral), implying a large sample phenotype heterogeneity. ASD is an etiologically heterogeneous disorder, and factors other than genetics act synergistically and converge to common biological pathways representing “core” mechanisms for disease development. However, different etiologies may result phenotypically in the wide spectrum of ASD symptoms. This study did not examine the dietary habits and stress levels of ASD-affected individuals. Dietary habits may influence metabolic health, and stress may affect an individual’s immune system and these merit further study.

## 5. Conclusions

Our main conclusion was that inflammatory cells and metabolic markers (triglycerides, HDL-C, LDL-C, cholesterol, and glucose) were within the normal range for the pediatric population (according to the CDC) in Brazilian children/adolescents with ASD. Also we demonstrated that older Brazilian children with ASD have higher values for triglycerides, insulin levels, free fatty acids, and the HOMA-index indicating a higher risk for insulin resistance. These markers can potentially be used as good tools to follow up the management of patients with ASD from their earliest childhood.

## Figures and Tables

**Table 1:** Characterization of ASD participants.

Characteristics	Number of Participants
Children/Adolescents	118/36
Males/Females	140/14
ASD Level 1	104
ASD Level 2	36
ASD Level 3	14
Underweight	2
Normal Weight	64
Overweight	24
Obese	64

**Table 2:** Marginal means and uncertainty estimates (90% credible intervals) of inflammatory cells in children and adolescents with ASD.

	Leukocyte (Cells/mm^3^)	Lymphocyte (Cells/mm^3^)	Monocyte (Cells/mm^3^)
Age group			
2 to 5 years	6327.3 (4519.2; 7993.2)	2845.1 (1784.4; 3780.3)	432.8 (203.7; 717.6)
6 to 9 years	6543.6 (4782.9; 8108.3)	2842.9 (1801.6; 3750.5)	395.9 (181.8; 669.1)
10 to 13 years	6594.2 (4851.1; 8184.4)	2806.6 (1759.3; 3726.4)	470.9 (253.9; 758.3)
14 to 17 years	6299.6 (4402.4; 7991.7)	2788.0 (1719.2; 3750.9)	405.1 (153.6; 695.1)
ASD Intensity			
Level 1	6426.3 (4948.0; 7851.0)	2707.5 (1866.3; 3455.1)	384.7 (175.2; 644.0)
Level 2	6816.2 (5300.7; 8302.9)	3148.6 (2297.4; 3943.4)	436.9 (222.6; 699.2)
Level 3	6081.0 (4026.5; 7953.3)	2605.9 (1416.2; 3631.2)	456.9 (188.3; 780.8)
Medication			
No	6459.3 (4610.6; 8146.6)	2783.8 (1731.3; 3738.9)	426.2 (182.9; 712.9)
Yes	6423.1 (4628.0; 7999.4)	2857.6 (1799.9; 3764.9)	426.1 (202.0; 714.5)
Gender			
Female	6391.9 (4489.9; 8110.0)	2669.3 (1598.1; 3636.9)	494.3 (245.1; 776.2)
Male	6490.5 (4769.3; 8039.8)	2972.0 (2065.6; 3830.5)	358.0 (162.1; 578.4)
Weight status			
Underweight	6482.4 (4520.8; 8334.7)	2919.0 (1830.6; 3944.7)	437.9 (185.0; 737.5)
Normal weight	5977.8 (4297.8; 7376.6)	2522.5 (1534.5; 3324.9)	394.5 (171.2; 671.2)
Overweight	6438.5 (4729.5; 7921.1)	2824.0 (1827.8; 3664.1)	442.5 (214.6; 729.4)
Obese	6866.0 (5169.4; 8290.5)	3017.1 (2028.3; 3841.1)	429.8 (205.1; 708.6)

**Table 3:** Marginal means and uncertainty estimates (90% credible intervals) of the metabolic profile indicators in children and adolescents with ASD.

	Glucose (mg/dL)	Insulin (µU/mL)	HOMA-IR
Age group			
2 to 5 years	73.4 (65.9; 79.7)	5.95 (0.96;11.34)	1.18 (0.19; 2.25)
6 to 9 years	75.2 (67.9; 81.3)	7.01 (2.17; 12.17)	1.36 (0.38; 2.39)
10 to 13 years	74.7 (67.3; 81.1)	8.64 (3.92; 13.86)	1.64 (1.63; 2.67)
14 to 17 years	74.5 (66.4; 81.9)	10.24 (4.74; 16.00)	1.97 (0.87; 3.12)
ASD Intensity			
Level 1	74.8 (67.5; 80.9)	7.40 (1.87; 13.29)	1.44 (0.36; 2.59)
Level 2	74.8 (67.4; 81.3)	7.97 (2.39; 13.92)	1.55 (0.47; 2.72)
Level 3	73.8 (65.6; 81.0)	8.50 (2.59; 15.00)	1.62 (0.46; 2.90)
Medication			
No	74.1 (66.3; 80.9)	8.31 (2.54; 14.43)	1.61 (0.37; 2.68)
Yes	74.9 (67.3; 81.2)	7.61 (2.03; 13.78)	1.47 (0.48; 2.81)
Gender			
Female	73.3 (65.5; 80.3)	8.22 (2.36; 14.46)	1.61 (0.45; 2.83)
Male	75.6 (69.0; 81.6)	7.69 (2.18; 13.73)	1.47 (0.40; 2.64)
Weight status			
Underweight	73.4 (64.0; 81.5)	5.67 (0.40; 11.19)	1.06 (0.05; 2.15)
Normal weight	72.9 (66.4; 78.4)	6.31 (2.34; 10.88)	1.22 (0.46; 2.10)
Overweight	75.6 (68.7; 81.6)	8.33 (4.19; 13.19)	1.63 (0.83; 2.58)
Obese	75.9 (69.4; 81.5)	11.52 (7.47; 16.25)	2.24 (1.48; 3.17)

**Table 4:** Marginal means and uncertainty estimates (90% credible intervals) of lipid profile indicators in children and adolescents with ASD.

	Cholesterol (mg/dL)	Triglycerides (mg/dL)	HDL-C (mg/dL)	LDL-C (mg/dL)	Fatty Acids (mmol/L)
Age group					
2 to 5 years	162.7 (140.6; 181.4)	82.6 (24.4; 140.9)	52.4 (42.6; 61.8)	90.7 (72.5; 105.9)	1.84 (−1.42; 5.81)
6 to 9 years	168.0 (146.7; 185.9)	93.6 (38.8; 150.6)	50.3 (41.0; 59.1)	99.6 (82.3; 114.0)	1.62 (−1.65; 5.56)
10 to 13 years	166.6 (144.8; 181.4)	131.3 (73.4; 191.5)	48.1 (38.1; 57.0)	93.7 (75.5; 109.1)	1.51 (−1.77; 5.44)
14 to 17 years	166.1 (142.2; 187.7)	162.2 (80.3; 249.9)	46.6 (33.8; 57.6)	91.4 (69.1; 110.4)	2.75 (−0.61; 6.65)
ASD Intensity					
Level 1	166.8 (145.4; 184.9)	112.8 (39.9; 202.2)	48.6 (37.6; 58.2)	96.9 (78.7; 112.5)	0.53 (−1.67; 2.14)
Level 2	166.9 (145.1; 185.9)	122.9 (49.0; 214.7)	49.5 (38.5; 59.4)	93.4 (74.2; 109.8)	0.46 (−1.76; 2.10)
Level 3	163.9 (140.0; 185.0)	116.5 (39.5; 209.0)	49.9 (38.4; 60.5)	91.3 (70.0; 109.4)	4.80 (2.65; 6.60)
Medication					
No	164.7 (142.0; 185.0)	118.0 (41.9; 210.2)	48.9 (37.7; 59.2)	91.9 (71.5; 109.3)	1.85 (−1.56; 5.96)
Yes	167.1 (144.8; 185.6)	116.8 (42.9; 207.6)	49.7 (38.6; 59.6)	95.8 (76.4; 111.8)	2.01 (−1.39; 6.10)
Gender					
Female	162.5 (139.4; 183.0)	115.3 (37.5; 207.7)	47.6 (36.5; 58.0)	92.7 (71.6; 110.8)	1.81 (−1.61; 5.91)
Male	169.3 (149.8; 186.8)	119.5 (47.8; 209.9)	51.0 (40.9; 60.3)	95.0 (76.2; 110.7)	2.05 (−1.33; 6.14)
Weight status					
Underweight	162.8 (135.1; 186.6)	112.5 (25.5; 208.8)	49.4 (36.6; 61.5)	91.9 (68.4; 111.1)	0.45 (−2.17; 4.16)
Normal weight	161.3 (142.3; 177.3)	103.2 (44.5; 185.3)	50.4 (41.3; 57.8)	91.5 (73.2; 106.7)	2.49 (0.31; 6.26)
Overweight	169.2 (149.0; 186.9)	103.0 (38.4; 187.4)	52.4 (42.4; 61.0)	96.2 (76.6; 112.8)	2.46 (0.26; 6.24)
Obese	170.3 (151.1; 186.6)	151.1 (91.4; 235.4)	45.2 (35.9; 52.7)	95.9 (77.4; 111.1)	2.33 (0.14; 6.11)

## Data Availability

The raw data supporting the conclusions of this article will be made available by the authors on written request. Please submit a written request to the authors for the raw and/or processed data files. The data are not publicly available due to privacy.
